# From bad to worse: a case report of bilateral iatrogenic coronary dissection complicated with cardiac arrest

**DOI:** 10.1093/ehjcr/ytag337

**Published:** 2026-05-08

**Authors:** Hamza Chraibi, Said Ouadallah, Sihem M’barki, Taha Hassani

**Affiliations:** Cardiology Department, Ibn Sina University Hospital, Mohammed V University, United Nations Avenue, Rabat 10000, Morocco; Cardiology Department, Ibn Sina University Hospital, Mohammed V University, United Nations Avenue, Rabat 10000, Morocco; Cardiology Department, Ibn Sina University Hospital, Mohammed V University, United Nations Avenue, Rabat 10000, Morocco; Cardiology Department, Jacques Puel Hospital, Hospital Avenue, Rodez 12027, France

**Keywords:** Coronary artery dissection, Cardiac arrest, Acute coronary syndrome, Percutaneous intervention, Case report

## Abstract

**Background:**

Iatrogenic coronary artery dissection is a rare but life-threatening complication, occurring in fewer than 0.1% of coronary procedures. It is typically precipitated by aggressive or deep intubation with large catheters. While most dissections are unilateral, bilateral occurrences are exceptionally rare and often suggest underlying ‘vulnerable’ vessel conditions, such as fibromuscular dysplasia.

**Case summary:**

A 40-year-old woman with no medical history presented with non-ST-elevation acute coronary syndrome. Initial angiography of the right coronary artery (RCA) and left main coronary artery (LMCA) showed no obstructive lesions. Post-procedure, the patient developed recurrent chest pain and circumferential ST-segment elevation, followed by ventricular fibrillation that required electrical cardioversion. A second angiogram revealed simultaneous dissections of both the LMCA and RCA. Emergent stenting was successfully performed to seal the dissection planes in both vessels. An extensive autoimmune and systemic vascular workup was negative. The patient recovered well and was discharged four days later.

**Discussion:**

LMCA dissections carry a high mortality risk because they jeopardize a vast territory of the myocardium. In this case, the simultaneous involvement of both the RCA and LMCA represents an extreme clinical challenge. Management must be immediate; urgent stenting is the gold standard to prevent total vessel occlusion, myocardial infarction, and irreversible cardiac damage.

Learning points

- Clinical instability or new ST-segment changes following a ‘normal’ angiogram must be treated as a procedural complication until proven otherwise.
- Simultaneous bilateral iatrogenic coronary artery dissection is an exceptionally rare complication that requires immediate recognition and emergent stenting.
- The occurrence of multi-vessel dissections in the absence of aggressive manipulation should prompt a comprehensive investigation for underlying vascular vulnerabilities.

## Introduction

Iatrogenic coronary artery dissection (ICAD) is a rare but potentially catastrophic complication of cardiac catheterization, occurring in fewer than 0.1% of diagnostic and interventional procedures. While the majority of these events are unilateral and involve a single vessel, simultaneous bilateral involvement is an exceptionally rare clinical phenomenon.^1–3^ The clinical spectrum ranges from localized, asymptomatic intimal tears to extensive dissections that cause acute vessel occlusion, myocardial infarction, and sudden cardiac death.

## Summary figure

**Figure:**
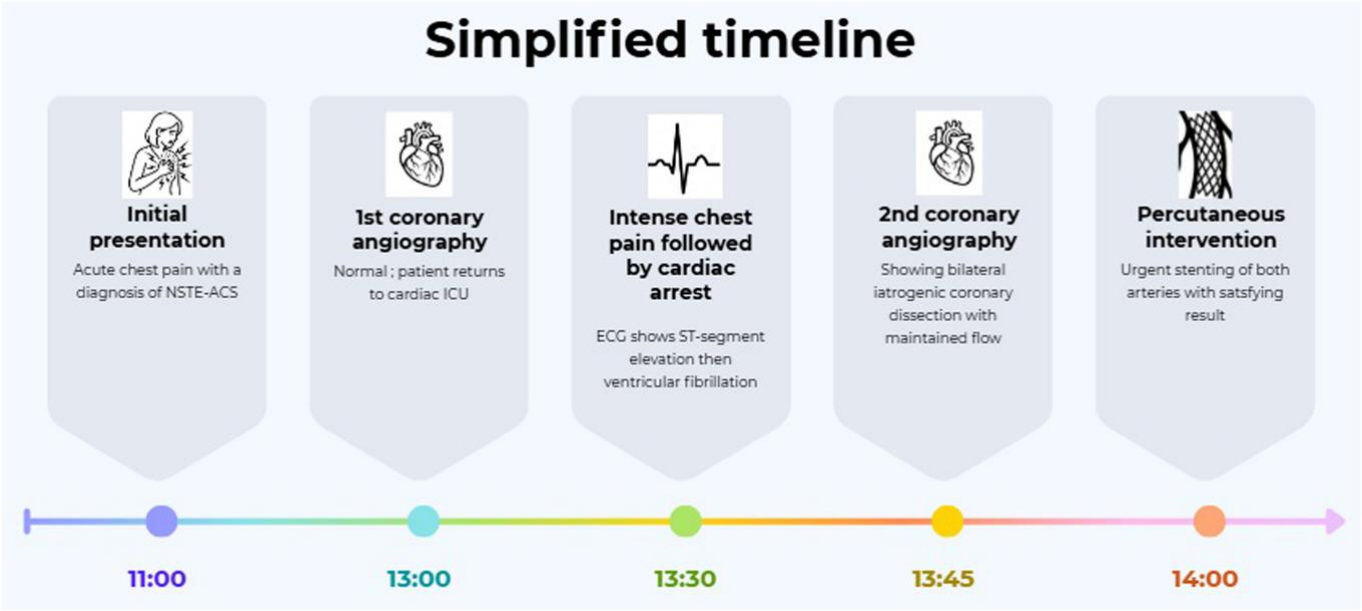


In this article, we present an exceptional case of bilateral iatrogenic coronary dissection complicated with cardiac arrest in a young patient with no coronary dissection. To our knowledge, this is the first case described in literature.

## Case presentation

A 40-year-old woman with no significant prior medical history or cardiovascular risk factors presented to the emergency department with an acute chest pain. Electrocardiogram (ECG) was normal, and high-sensitivity troponin levels were very elevated, rising in less than 3 h from 900 to 8000 ng/L. An echocardiogram done in the emergency department found hypokinesia of the left ventricle’s lateral wall. Because of these findings, and the high likelihood of non-ST-elevation acute coronary syndrome (NSTE-ACS), the patient was fast-tracked for an urgent coronary angiography as protocol.

**Figure 1:**
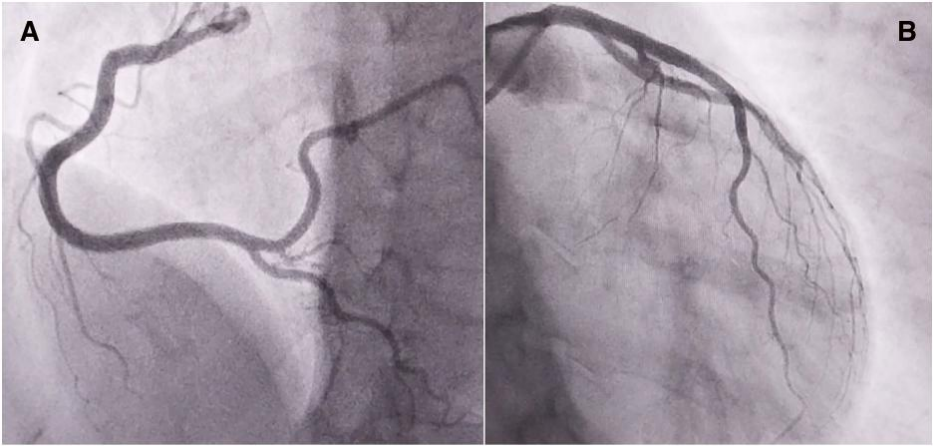
Initial coronary angiogram performed with JR4.0 5 F and EBU 3.5 6 F catheter showing normal right (*A*) and left (*B*) coronary arteries.

The procedure, using right radial access, began with a moderately deep engagement of the right coronary artery (RCA) using a JR4.0 5 F diagnostic catheter, which revealed smooth vessel walls and no obstructive lesions (*Figure 1A*, Supplementary material online, *Video S1*). Subsequently, because the operator believed there was a lesion on the left main coronary artery (LMCA) or its branches, and in the interest of saving time, they engaged it using an EBU 3.5 6 F catheter. This also demonstrated a normal vessel with TIMI 3 flow and no evidence of atherosclerosis or dissection at that time (*Figure 1B*, Supplementary material online, *Video S4*).

**Figure 2:**
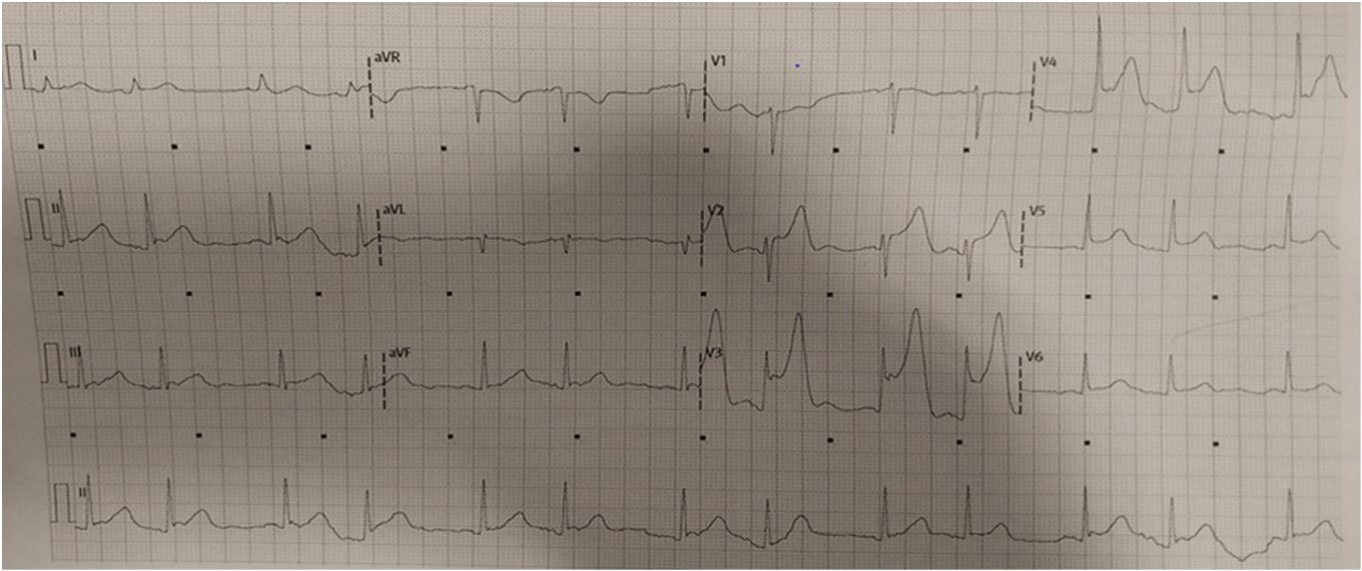
Electrocardiogram performed after the coronary angiography showing diffuse ST-segment elevation.

Shortly after returning to the intensive care unit, the patient’s condition deteriorated rapidly as she reported a sudden onset of crushing chest pain. An immediate ECG showed circumferential ST-segment elevation, indicating global subendocardial ischaemia or multi-vessel involvement (*Figure 2*). Moments later, the patient collapsed into ventricular fibrillation.

**Figure 3:**
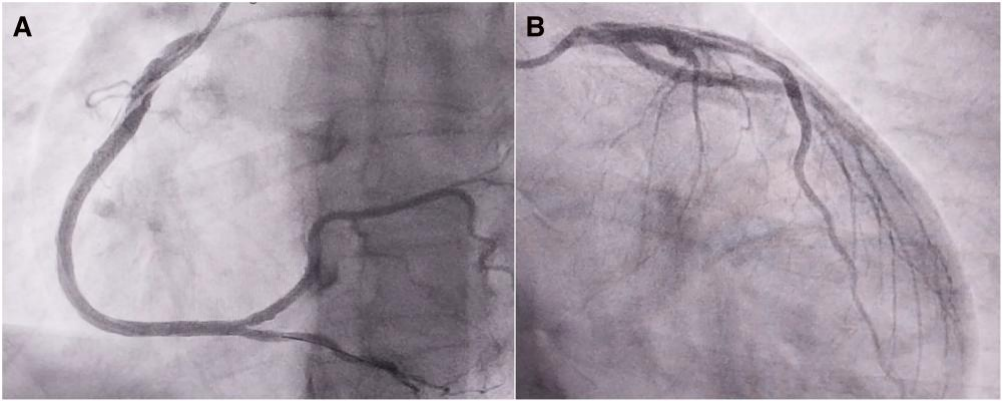
Second coronary angiogram showing a coronary dissection in both the right coronary artery (*A*) and the left main extending to the left anterior descending artery (*B*).

Advanced cardiac life support was initiated, and the patient was successfully resuscitated following electrical cardioversion. She was immediately rushed back to the catheterization laboratory. A repeat angiogram, using right radial access as well, revealed a double-vessel disaster: extensive, flow-limiting dissections involving both the RCA (*Figure 3A*, Supplementary material online, *Video S3*) and the LMCA extending to the left anterior descending (*Figure 3B*, Supplementary material online, *Video S4*).

**Figure 4:**
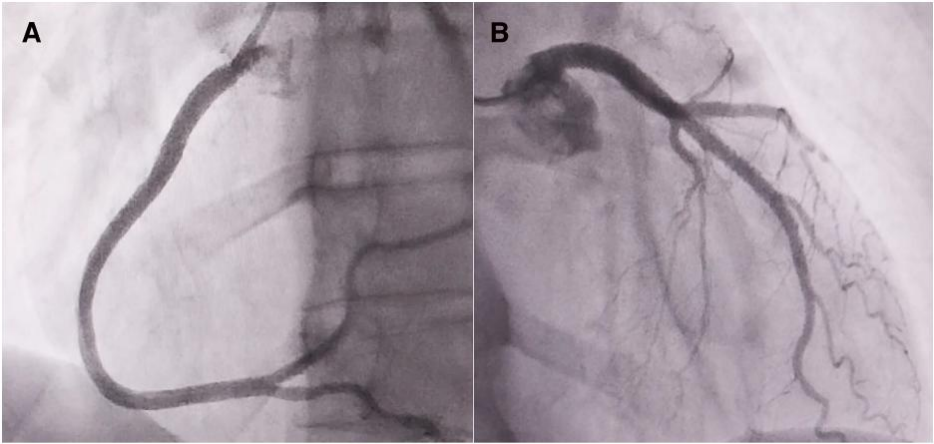
Control angiogram after stenting showing satisfactory results in the right (*A*) and left (*B*) coronary arteries.

To stabilize the patient, emergent percutaneous coronary intervention (PCI) was performed. Stents were deployed across the dissection planes in both arteries, successfully sealing the false lumens and restoring anterograde flow (*Figure 4A* and *B*).

Following the procedure, an extensive diagnostic workup was initiated to identify a systemic cause for such ‘vulnerable’ vessels. The autoimmune screening returned negative for vasculitis markers. Vascular imaging (CT angiography of the neck, abdomen, and pelvis) also returned negative.

The patient’s recovery was favourable. She remained haemodynamically stable and was discharged four days later on a regimen of dual antiplatelet therapy (DAPT).

## Discussion

The management of LMCA dissection represents one of the most high-stakes scenarios in interventional cardiology. Because the LMCA provides the primary blood supply to the majority of the left ventricular myocardium, any compromise in its patency can lead to rapid haemodynamic collapse.^4^ This case serves as a critical reminder that any sudden clinical or electrical instability following a coronary procedure must be treated as a procedural complication until proven otherwise.

Aggressive and deep intubation using large catheters is the main precipitating factor in coronary angiography. The graphical abstract in the supplemental data proposes a simplified classification of factors increasing the risk of iatrogenic coronary artery dissection.

In this specific case, the simultaneous involvement of the RCA and LMCA suggests that the vessels may have been predisposed to injury. Although the systemic workup was negative, the possibility of a localized vascular fragility cannot be entirely ruled out.^5,6^ The transition from a normal angiogram to a bilateral dissection highlights the ‘latent’ nature of some iatrogenic injuries, where the intimal flap may only propagate or cause occlusion minutes to hours after the initial trauma.

The ‘gold standard’ for managing iatrogenic dissection is immediate stenting. Goals of the intervention are to seal the entry point, restore TIMI 3 flow, and prevent propagation.^7^

## Supplementary Material

ytag337_Supplementary_Data

## Data Availability

The data underlying this article are available in the article and its online Supplementary material.

## Lead author biography

Dr. Hamza Chraibi is a young attending cardiologist from Morocco, currently working at the Mohammed VI Foundation of Science and Health in Rabat. He specializes in interventional cardiology and holds a passion for research and scientific innovation.

## Notes

**Consent:** Written informed consent for publication was obtained from the patient in line with the COPE guidance.
